# Degree of fear of needles and preferred allergy immunotherapy treatment among children with allergic rhinitis: caregiver survey results

**DOI:** 10.3389/fped.2024.1447619

**Published:** 2024-08-02

**Authors:** Karen Rance, Michael Blaiss, Payel Gupta, Hendrik Nolte, Erin P. Scott, Donna D. Gardner

**Affiliations:** ^1^ ALK, Bedminster, NJ, United States; ^2^ Department of Pediatrics, Medical College of Georgia, Augusta, GA, United States; ^3^ Mount Sinai Hospital, New York, NY, United States; ^4^ Scott Medical Communications, LLC, Tyler, TX, United States; ^5^ Allergy & Asthma Network, Fairfax, VA, United States

**Keywords:** allergy immunotherapy, injections, sublingual, oral, needle, preference, fear, allergic rhinitis

## Abstract

**Introduction:**

A child's fear of needles may impact the preferred route of allergy immunotherapy (AIT) when choosing between subcutaneous immunotherapy (allergy shots) or sublingual immunotherapy (SLIT). A survey was conducted to understand caregiver health-seeking behavior for children with allergic rhinitis with or without conjunctivitis (AR/C) and explore if fear of needles impacted AIT decisions.

**Methods:**

Caregivers of children ages 5–17 years with AR/C were recruited from the Dynata US research panel to participate in an online survey from May-June 2023. The survey received institutional review board exemption status. SLIT-tablets were described as “under-the-tongue tablets”.

**Results:**

About a third (34%) of surveyed caregivers (*n* = 437) reported their child had a severe fear of needles and 47% reported moderate fear. Of surveyed caregivers, 53% and 43% reported they had discussed allergy shots and SLIT-tablets, respectively, with their child's physician. SLIT-tablets were preferred by 84% of caregivers; 6% preferred injections and 10% had no preference. Caregivers of children with a severe fear of needles had the highest preference for SLIT-tablets (95%) vs. injections (2%); 85% and 60% of caregivers of children with moderate and low fear, respectively, preferred SLIT-tablets. Among caregivers of children with a severe fear of needles, a higher percentage agreed that their child would welcome taking SLIT-tablets than that their child would accept taking an ongoing series of allergy shots (93% vs. 43%, respectively).

**Conclusions:**

Most caregivers preferred SLIT-tablets over allergy shots for their child with AR/C. Preference for SLIT-tablets corresponded with the child's degree of fear of needles. Fear of needles should be included in AIT shared decision-making conversations.

## 1 Introduction

Allergic rhinitis with or without conjunctivitis (AR/C) is one of the most common childhood diseases, affecting 18% of children worldwide (1). The symptoms of AR/C, namely sneezing, nasal congestion, rhinorrhea, itchy nose, and itchy eyes, can interfere with sleep and daily activities (2, 3). Subsequently, children with AR/C can experience fatigue, poor concentration, and feel irritable, unhappy, angry, and embarrassed (2). The presence of AR/C symptoms can also have a negative effect on cognitive function and performance on school exams (4, 5). Furthermore, AR/C is a risk factor for the development of asthma (6).

Management of AR/C typically includes allergen avoidance measures, symptom-relieving pharmacotherapy (e.g., antihistamines, intranasal corticosteroids, and intranasal decongestants), or allergy immunotherapy (AIT) (7). AIT differs from pharmacotherapy in that it modifies the immune response and induces tolerance to the allergen to which the patient is sensitized. A 3-year regimen of AIT can result in long-term reductions in AR/C symptoms and pharmacotherapy use for years after the end of the treatment period (8–11). AIT in children can also reduce the risk of developing asthma and decrease asthma symptoms and medication use in patients with existing allergic asthma (12, 13). Subcutaneous immunotherapy (SCIT, aka “allergy shots”) or sublingual immunotherapy (SLIT)-tablets are approved modes of AIT administration. SCIT is generally administered every 2 to 4 weeks in a physician's office once the maintenance dose is reached (14). SLIT-tablets are an injection-free AIT option administered daily at home after the first dose has been taken and shown to be tolerated in a clinical setting (15).

SCIT can be initiated in children of any age at the discretion of the physician, although it is generally not advised for infants and toddlers (14). Grass and ragweed SLIT-tablets are approved for children ages 5 years and up, and the house dust mite SLIT-tablet is approved for ages 12 years and up (14, 15). By the age of 5 years, most children have received vaccinations and know that injections can cause pain. Anxiety and fear in children caused by the anticipation of injection-related pain is a well-known phenomenon (16, 17). Thus, a child's fear of needles may impact the preferred route of AIT when choosing between SCIT or SLIT-tablets. A survey was conducted to understand caregiver health-seeking behavior for children with AR/C and explore if fear of needles impacted AIT decisions.

## 2 Methods

### 2.1 Survey eligibility and methodology

Members of the Dynata US research panel were recruited by an electronic invitation to participate in a cross-sectional online survey from May-June 2023. Dynata is a market research firm. Eligible participants were caregivers in the US aged 21 years or older, of children ages 5–17 years currently being treated by a health professional for “environmental allergies”, “allergic rhinitis”, “hay fever”, “allergic conjunctivitis”, or “food allergy”. For the purposes of the current report, “AR/C” will be used to represent all the terms except “food allergy”. The survey specified that if any participants had more than 1 child with these conditions, they should only consider and report on one of the children throughout the survey.

The survey received institutional review board exemption status. Individuals provided consent to participate in the survey. All individuals recruited for the survey were assigned an ID number to ensure confidentiality and unauthorized access. Each individual was allowed to participate only once in the survey. To complete the survey, participants were given an incentive of panel points toward earning financial compensation. Recommendations from the Consensus-Based Checklist for Reporting of Survey Studies were followed in the current report (18).

Pretesting of the survey was conducted within the programming and project management teams at Dynata and Allergy & Asthma Network, followed by a soft launch of 10% of the survey completion goal.

### 2.2 Survey characteristics

The survey contained 2 parts. Part 1 included 10 questions for caregivers of children with AR/C related to fear of needles and AIT (Supplementary Table S1). Part 2 of the survey was specific for caregivers of children with food allergy, the results of which are not included in the current report.

The following definitions were given to survey participants:

“Needle phobia: A patient is described as having needle phobia when they describe or display an intense fear or anxiety when they see a needle, or they need an injection for a medical procedure.”
“Allergic reaction: An abnormal mild, moderate, or severe response of the immune system to certain foreign substances. This is caused by hypersensitivity of the immune system to certain allergens that may come in contact with the body through breathing them in from the air, eating and digesting them, or coming in contact with the skin.”
“Hay fever: Also called allergic rhinitis, occurs when the body's immune system reacts to normally harmless things in the environment. Something in the environment that causes an allergic reaction is called an allergen. It could be grass, tree or ragweed pollen, mold, or pet dander. Common symptoms of hay fever upon exposure to an allergen are runny nose, usually with clear or pale-colored mucus, sneezing, coughing, red, watery eyes, and itching around the nose, mouth or eyes.”
“Allergy immunotherapy (AIT): A treatment option for hay fever, environmental allergies, and allergic asthma. AIT helps build a patient's tolerance to allergens, reducing or eliminating symptoms. The patient is repeatedly given small but increasing doses of the allergen on a regular schedule for 3–5 years. For many patients, symptoms are reduced or eliminated even after AIT ends. AIT is given through shots, under-the-tongue dissolvable tablets, or under-the-tongue drops.”
“Allergic conjunctivitis: Eye infection.”

SLIT-tablets were described as “under-the-tongue tablets”, and SCIT was described as “allergy shots” or “injection” in survey questions.

Questions regarding the severity of the fear of needles were scored on a scale of 0 (no fear), 1 (minimal or low fear), 2, 3, 4, and 5 (severe or life-altering fear). A score of 0 or 1 was considered low fear, 2 or 3 was considered moderate fear, and 4 or 5 was considered severe fear. Questions regarding agreement or likelihood were scored on a scale of 1 (disagree strongly or very unlikely), 2, 3, 4, and 5 (agree strongly or very likely). A score of 4 or 5 was considered agreement or likely.

### 2.3 Analysis

The participation goal was 500 surveys completed by individuals that were representative of the general US population in terms of age, race (10%–15% Black), ethnicity (5%–10% Hispanic), education, and household income. Analysis of survey responses was descriptive only and was conducted in Excel. Categorical questions are reported as the percentage of survey participants reporting responses.

## 3 Results

Of the 3,084 surveys initiated, 1,893 caregivers did not pass the eligibility screening, and 690 surveys were incomplete; 501 caregivers completed the survey, and 437 were caregivers of children with AR/C. Of these 437 caregivers, 59% identified as female, 74% identified as White, 15% identified as Black, and 19% identified as Hispanic (Table 1). A slight majority (55%) of the children had ever received AIT in the form of SCIT, SLIT drops, or SLIT-tablets.

**Table 1:** Surveyed caregiver characteristics.

Characteristic	Surveyed caregivers *N* = 437
Female, %	59
Age, years, %	
21–34	29
35–44	38
45+	33
Race, %	
American Indian/Alaska Native	2
Asian	4
Black	15
Multiracial	2
Native Hawaiian or Pacific Islander	1
White	74
Other	2
Ethnicity	
Hispanic/Latino, %	19
Annual household income, %	
Low (<$50,000)	29
Middle ($50,000–99,999)	41
High ($100,000+)	30

**Figure 1:**
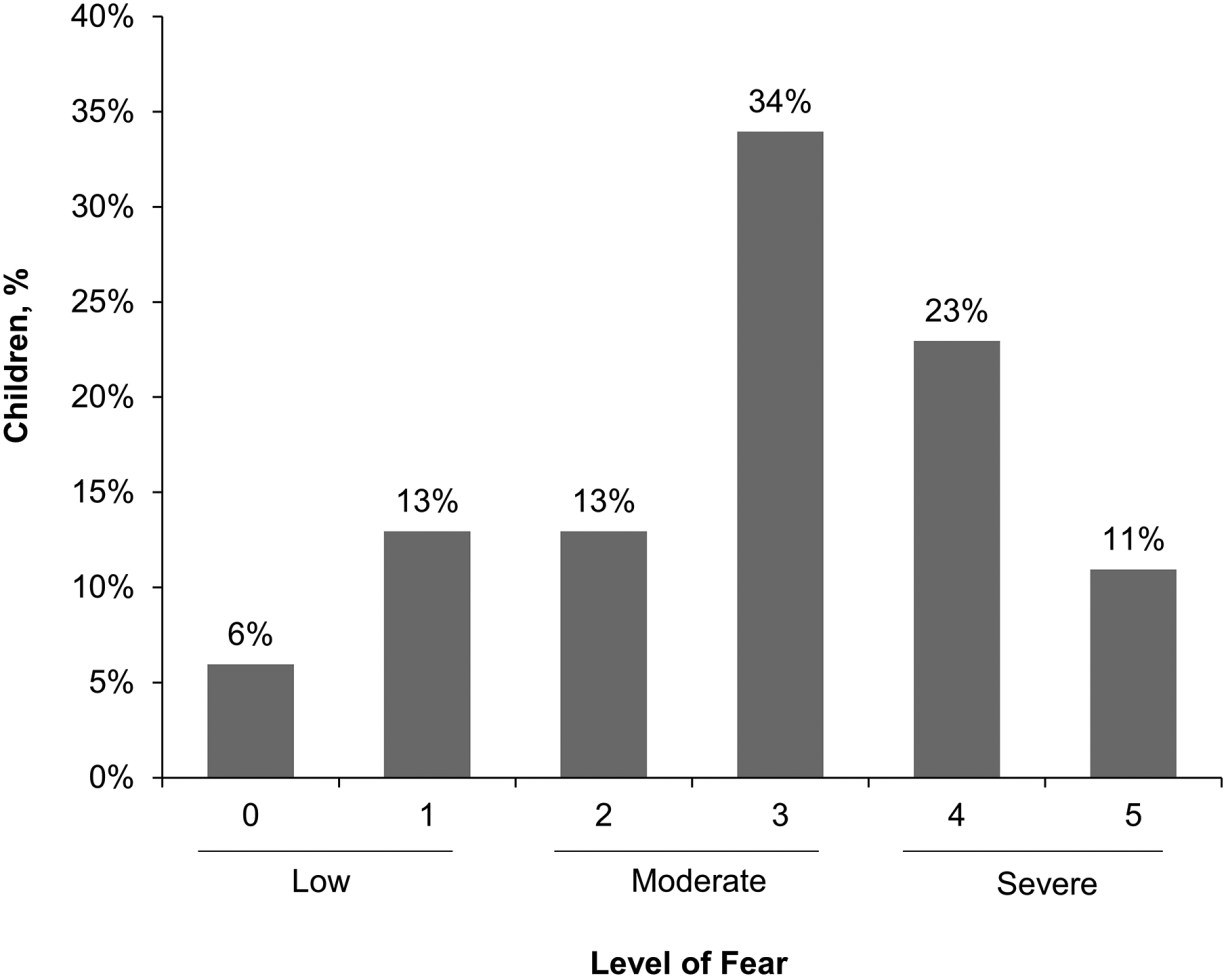
Caregiver reported severity of child's fear of needles. Scale was 0 (no fear), 1 (minimal or low fear), 2, 3, 4, or 5 (severe or life-altering fear).

**Figure 2:**
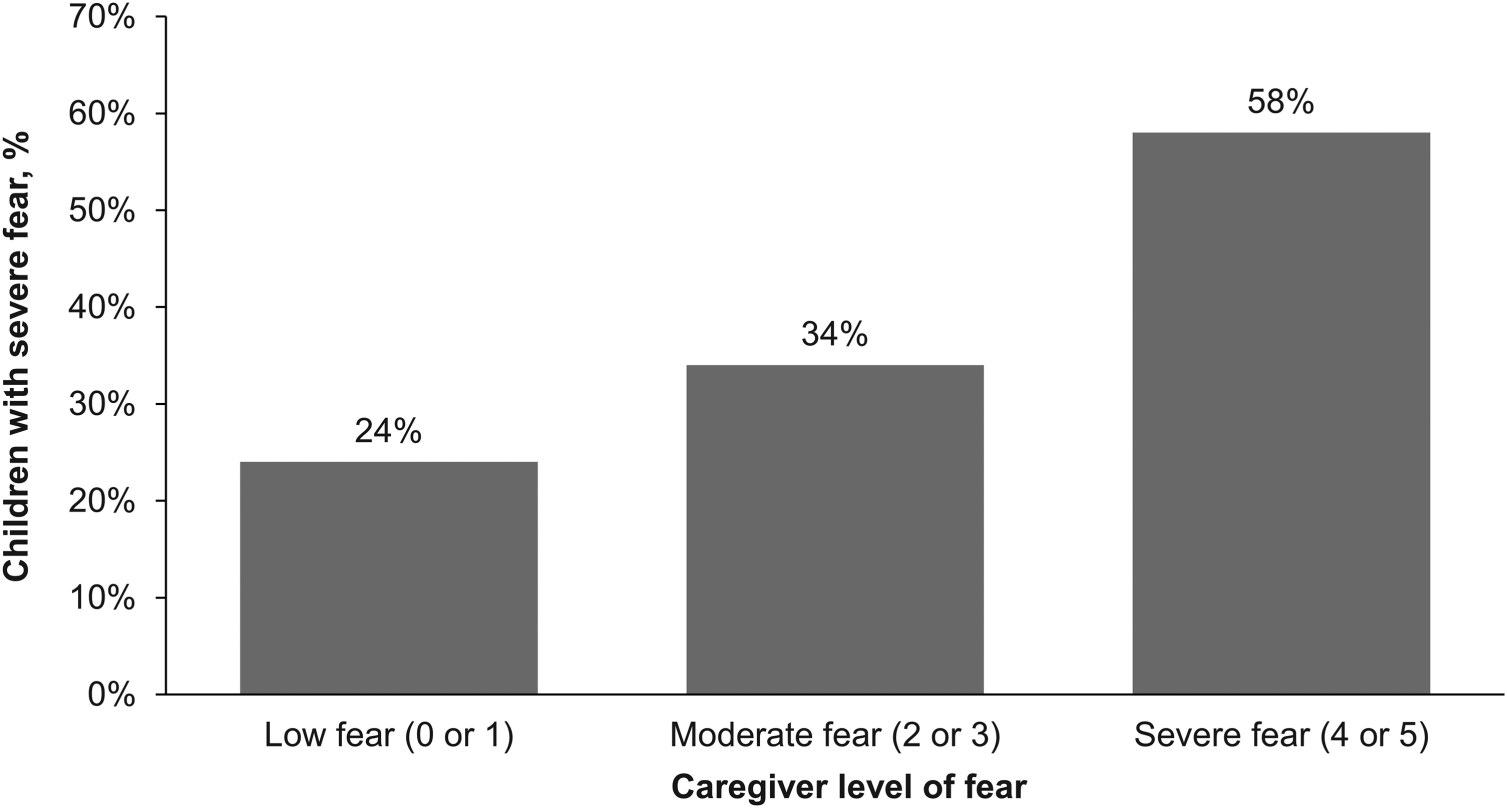
Percentage of children with severe fear of needles by caregiver's level of fear. Scale was 0 (no fear), 1 (minimal or low fear), 2, 3, 4, or 5 (severe or life-altering fear).

About a third (34%) of caregivers reported their child had a severe fear of needles (score of 4 or 5) and 47% reported moderate fear (score of 2 or 3; Figure 1). Only 19% reported their child had low fear (score of 0 or 1). Adolescents aged 12–17 years (*n* = 186) were less likely to have a severe fear of needles than children aged 5–11 years (*n* = 251; 29% vs. 38%, respectively). Caregivers also reported similar levels of fear for the child's siblings (28% severe fear, 49% moderate fear, and 23% low fear). Approximately one in five (21%) caregivers reported their own fear of needles was severe whereas 47% reported low fear. There was an association between a child's level of fear of needles and their caregiver's level of fear; caregivers who were most fearful had the highest percentage of children who had a severe fear (Figure 2).

Caregivers reported that their child's level of fear of needles had not changed over the last 2–3 years for 52% of children, whereas fear had increased in 25% of the children and decreased in 23%; 25% of adolescents ages 12–17 years had decreased fear compared with 21% of children ages 5–11 years.

**Figure 3:**
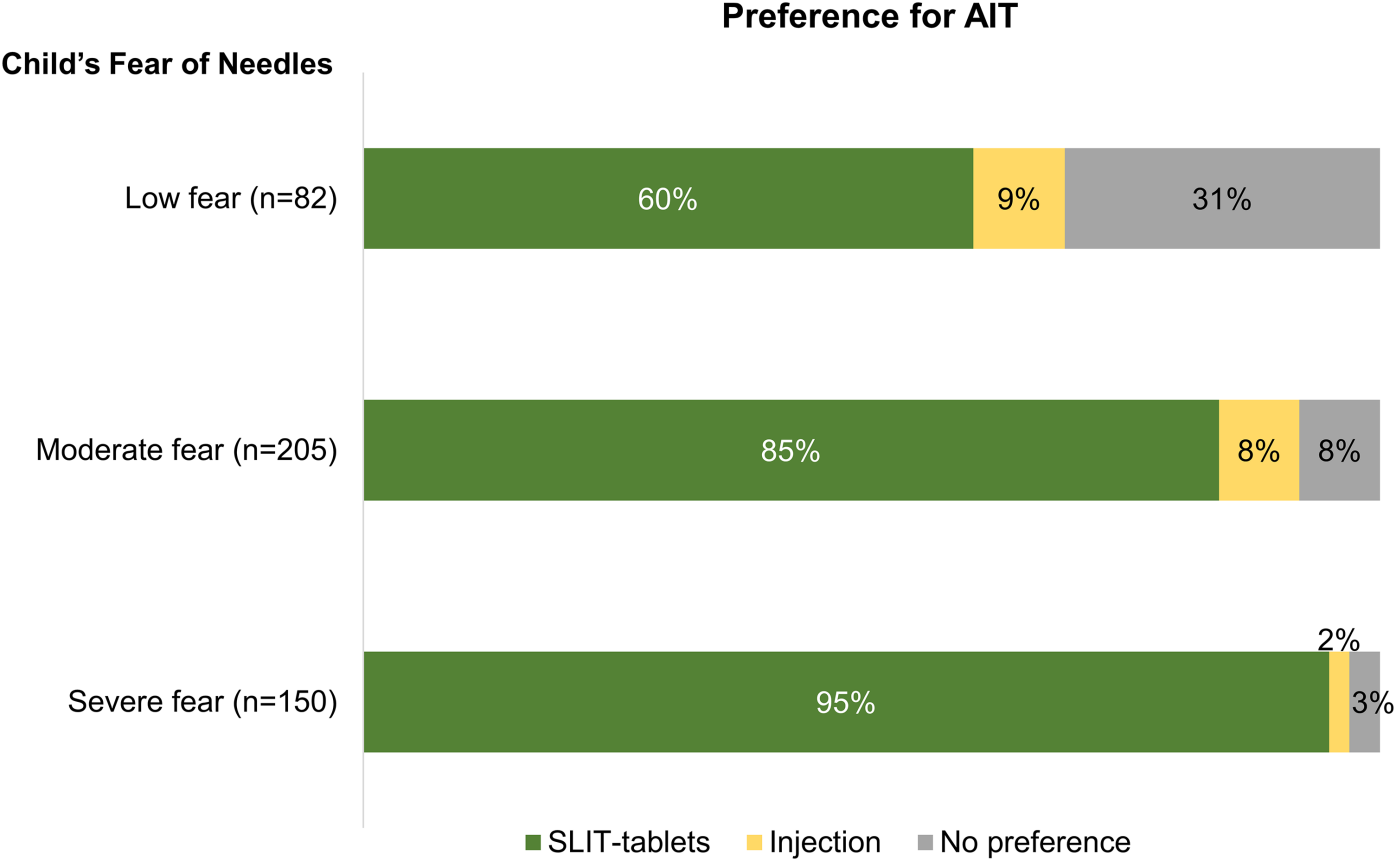
Percentage of caregiver responses to AIT preferences for their child by the caregiver's perception of their child's level of fear of needles.

Of surveyed caregivers, 53% and 43% reported they had discussed allergy shots and SLIT-tablets, respectively, with their child's physician. SLIT-tablets were preferred by 84% of caregivers; 6% preferred injections and 10% had no preference. Caregivers of children with a severe fear of needles had the highest preference for SLIT-tablets (95%) vs. injections (2%); 85% and 60% of caregivers of children with moderate and low fear, respectively, preferred SLIT-tablets (Figure 3). Overall, 85% of caregivers agreed their child would welcome taking SLIT-tablets, and 38% agreed their child would accept taking an ongoing series of allergy shots. Among caregivers of children with a severe fear of needles, a higher percentage agreed that their child would welcome taking SLIT-tablets than that their child would accept taking an ongoing series of allergy shots (93% vs. 43%, respectively; Table 2).

**Table 2:** Caregiver responses to child's acceptance of AIT treatment and caregiver likelihood of giving their child AIT treatment by the caregiver's perception of their child's level of fear of needles.

Survey question	Caregiver's perception of their child's fear of needles		
	Low (*n* = 82)	Moderate (*n* = 205)	Severe (*n* = 150)
My child would welcome taking an under-the-tongue dissolvable tablet every day for AIT when indicated and appropriate	72%	84%	93%
My child would accept taking an ongoing series of allergy shots for AIT when indicated and appropriate	54%	26%	43%
Likely to give their child AIT that requires taking an under-the-tongue dissolvable tablet every day at home	79%	83%	96%
Likely to bring their child to the doctor's office weekly or every other week for AIT	55%	55%	81%

AIT, allergy immunotherapy.

Most (87%) caregivers indicated they would be likely to give under-the-tongue tablets daily at home to their children with AR/C, and 64% indicated they would be likely to bring their children with AR/C weekly or every other week to the doctor's office to obtain AIT. Of caregivers of children with a severe fear of needles, 96% indicated they were likely to give under-the-tongue tablets at home and 81% indicated they were likely to bring their children to weekly or every other week AIT appointments (Table 2).

## 4 Discussion

The benefits of AIT in the treatment of AR/C are well established (19, 20), and in children there is the potential added benefit of the prevention of future asthma (12). Yet use of AIT in children is relatively low (21). Fear of receiving an injection when going to the doctor's office is common in children, and an AIT regimen that requires frequent (weekly or every other week) injections could be one reason that contributes to low pediatric participation with SCIT. In the current survey, over one-third of the caregivers indicated their child had a severe fear of needles. When given the choice between SCIT and a non-injection form of AIT (SLIT-tablets), most caregivers preferred SLIT-tablets for their child. The preference for SLIT-tablets corresponded with the child's degree of fear of needles; caregivers with children who had a severe fear of needles had the greatest preference for SLIT-tablets.

Previous surveys have shown that patients are more willing to try SLIT than SCIT (22, 23), although these surveys did not take fear of needles in children into consideration. In a survey of adults, fear of needles was given as a reason for not starting SCIT in only 6% of patients (23). The fear of needles appears to decrease with age since the percentage of those with a severe fear in the current survey decreased from 38% in children ages 5 to 11 years, to 29% in adolescents, to 21% in caregivers. Offering SLIT-tablets as an alternative to SCIT could encourage AIT uptake in children and adolescents by overcoming the barrier of fear of needles.

The current survey clearly indicates that fear of needles is an aspect of AIT that needs to be discussed among the prescriber, caregiver, and the child during shared-decision making conversations, but other factors also need to be discussed (24). Efficacy and safety are two key topics. Both SCIT and SLIT-tablets have been shown by meta-analyses to be effective in reducing AR/C symptoms (19, 20, 25), whereas SLIT has a more favorable safety profile than SCIT in terms of severe or life-threatening systemic allergic reactions (26). Cost and convenience are two other predominant factors for AIT that affect patient preference and subsequent adherence (23, 27). The convenience of AIT administration also appeared to be a factor for caregivers in the current survey, since a higher percentage reported they would be willing to have their child take a tablet every day at home for AIT than to bring their child to the doctor's office weekly or every other week for AIT.

One limitation of this survey is the potential for selection bias as all participants were members of a marketing research panel, which could impact the generalizability of the results. Another limitation is that the results relied upon the perceptions of the caregiver rather than responses from the children themselves. The caregiver's reported level of fear of needles for their child corresponded to that of their own level of fear, suggesting that the caregiver projected their own beliefs onto their child. Another possibility is that the caregiver's fear has been expressed to the child which then manifests as actual fear by the child. Another limitation is that the severity of the child's AR/C and the presence of polysensitization were not assessed, both of which are factors that can influence AIT decisions.

AIT is an effective treatment option for AR/C in children and adolescents, but uptake is relatively low. The results of this survey indicate that fear of needles is one possible barrier to initiating SCIT. Caregivers prefer SLIT-tablets as an alternative to SCIT, particularly those whose children have a severe fear of needles. This highlights the importance of having a shared decision-making conversation with the caregiver and child regarding the preferred route of AIT administration.

## Data Availability

The original contributions presented in the study are included in the article/supplementary material, further inquiries can be directed to the corresponding author/s.
