# Supplementary material for: Degree of fear of needles and preferred allergy immunotherapy treatment among children with allergic rhinitis: caregiver survey results
Source: Front Pediatr. 2024 Aug 2;12:1447619. doi: 10.3389/fped.2024.1447619 (PMC11327818; doi:10.3389/fped.2024.1447619)
Supplement: Supplementary file 1 [file Datasheet1.pdf]

Supplemental Material

Table S1. Survey questions.

1. What is your age? (Please enter a whole number)

2. With which of the following do you most identify?

    1. Male
    2. Female
    3. Non-binary
    4. Other
    5. Prefer not to answer

3. Do you consider yourself to be of Hispanic or Latino origin/culture? (Select one)

    1. Yes
    2. No

4. Which of the following best describes your racial identity? (Select one)

    1. White/Caucasian
    2. Black/African-American
    3. Asian/Asian-American
    4. American Indian or Alaska Native
    5. Native Hawaiian or other Pacific Islander
    6. Multiracial
    7. Other

5. What is the highest level of education that you have had the opportunity to complete? (Select one)

    1. Less than a high school degree
    2. High school degree or equivalent (e.g., GED)
    3. Some college but no degree
    4. Associate degree
    5. Bachelor’s degree
    6. Graduate degree, law degree, post-doctoral or doctoral degree
    7. Prefer not to answer

6. What is the 5-digit zip code of your primary residence?

7. Are you the parent or caregiver of a child under age 18?

    1. Yes
    2. No

8. For which of the following conditions, if any, is your child or are your children currently being treated by a healthcare professional? (Select all that apply)

    1. Hay fever
    2. Environmental allergies
    3. Allergic rhinitis
    4. Allergic conjunctivitis
    5. Food allergy
    6. Eczema
    7. Asthma
    8. Mononucleosis
    9. Cancer
    10. Other (Please specify)
    11. None of the above

9. Has your child ever been treated with allergy immunotherapy (AIT) in the form of shots or drops or tablets placed under the tongue? (If you have more than one child with these conditions, please think of, and answer for, just one of your children.)

    1. Yes
    2. No

10. How old is your child with hay fever, environmental allergies, allergic rhinitis, allergic conjunctivitis or a food allergy? (Again if you have more than one child with these conditions, please think of, and answer for, only one of your children.)

    1. 0-4
    2. 5-11
    3. 12-17
    4. 18 or older

11. How familiar are you with the concept of "needle phobia" when your child is being treated for hay fever, environmental allergies, allergic rhinitis, or allergic conjunctivitis? (Select one)

    1. Not at all familiar; I have never heard of needle phobia
    2. Familiar but my child's physician has not used the term needle phobia with me

3. Familiar but my child's physician has used the term needle phobia with me only once or twice
4. Familiar and my child's physician has used the term needle phobia with me occasionally
5. Familiar and my child's physician has used the term needle phobia with me frequently

12. How severe is your child's fear of needles? (Select one)

0 - My child has no fear of needles
1 – Minimal or low
2
3
4
5-Severe or life-altering

13. How severe is your own fear of needles? (Select one)

0 – I have no fear of needles
1 – Minimal or low
2
3
4
5-Severe or life-altering

14. How severe is your child's siblings' fear of needles? (If more than one sibling, please answer for the one most severe) Select one)

00 – My child has no siblings
0 - My child's siblings have no fear of needles
1 – Minimal or low
2
3
4
5-Severe or life-altering

15. How has your child's fear of needles changed over the last 2-3 years?

1. Fear has increased
2. Fear has not changed
3. Fear has decreased

16. Assuming that a tablet has the same intended health outcome, would you prefer under-the-tongue tablet treatment instead of injection for your child?

1. Yes – prefer under-the-tongue tablet treatment
2. No – prefer injection
3. No preference

17. Which of the following Allergy Immunotherapy (AIT) treatments for your child have you discussed with your child's doctor? (Select all that apply)

1. Allergy shots
2. Under-the-tongue tablets
3. Allergy drops in the mouth
4. Other (Please specify)
5. None of these

18. Please indicate your strength of agreement with the following statements.

	Disagree Strongly 1	2	3	4	Agree Strongly 5
My child would accept taking an ongoing series of allergy shots for Allergy Immunotherapy (AIT) when indicated and appropriate.					
My child would welcome taking an under-the-tongue dissolvable tablet every day for Allergy Immunotherapy (AIT) when indicated and appropriate.					

19. How likely are you to bring your child to the doctor's office weekly or every other week to obtain Allergy Immunotherapy (AIT)?

1-Very unlikely
2
3
4
5-Very likely

20. How likely are you to give your child Allergy Immunotherapy (AIT) that requires your child to take an under-the-tongue dissolvable tablet every day at home?

1-Very unlikely
2
3
4
5-Very likely

21. Which of the following best describes the neighborhood where you live? (Select one)

1. Major metropolitan area
2. Small city
3. Suburb
4. Rural area

22. In what state is your primary residence?

23. Which of the following best describes the type of health insurance coverage that you currently have for your child/children? (Select one)

1. Group private insurance obtained by me personally or through an employer or COBRA
2. Medicare
3. Medicaid
4. Other (please specify)
5. I don't have any health insurance coverage
6. I don't know

24. Approximately what was your annual gross household income last year before taxes (2022)?

1. $0 – $9,999
2. $10,000 – $19,999
3. $20,000 – $29,999
4. $30,000 – $39,999
5. $40,000 – $49,999
6. $50,000 – $59,999
7. $60,000 – $69,999
8. $70,000 – $79,999
9. $80,000 – $89,999
10. $90,000 – $99,999
11. $100,000 or more
12. Prefer not to answer
